# Wear patterns in knee OA correlate with native limb geometry

**DOI:** 10.3389/fbioe.2022.1042441

**Published:** 2022-11-18

**Authors:** A. Van Oevelen, I. Van den Borre, K. Duquesne, A. Pizurica, J. Victor, N. Nauwelaers, P. Claes, E. Audenaert

**Affiliations:** ^1^ Department of Orthopedic Surgery and Traumatology, Ghent University Hospital, Ghent, Belgium; ^2^ Department of Human Structure and Repair, Ghent University, Ghent, Belgium; ^3^ Department of Electromechanics, InViLab Research Group, University of Antwerp, Antwerp, Belgium; ^4^ TELIN-GAIM, Faculty of Engineering and Architecture, Ghent University, Ghent, Belgium; ^5^ Medical Imaging Research Center, MIRC, University Hospitals Leuven, Leuven, Belgium; ^6^ Department of Electrical Engineering, ESAT/PSI KU Leuven, Leuven, Belgium; ^7^ Department of Human Genetics, KU Leuven, Leuven, Belgium; ^8^ Murdoch Childrens Research Institute, Royal Children’s Hospital, Melbourne, VIC, Australia; ^9^ Department of Trauma and Orthopedics, Addenbrooke’s Hospital, Cambridge University Hospitals NHS Foundation Trust, Cambridge, United Kingdom

**Keywords:** statistical shape analysis, knee diagnostic imaging, osteoarthritis, alignment, knee wear

## Abstract

**Background:** To date, the amount of cartilage loss is graded by means of discrete scoring systems on artificially divided regions of interest (ROI). However, optimal statistical comparison between and within populations requires anatomically standardized cartilage thickness assessment. Providing anatomical standardization relying on non-rigid registration, we aim to compare morphotypes of a healthy control cohort and virtual reconstructed twins of end-stage knee OA subjects to assess the shape-related knee OA risk and to evaluate possible correlations between phenotype and location of cartilage loss.

**Methods:** Out of an anonymized dataset provided by the Medacta company (Medacta International SA, Castel S. Pietro, CH), 798 end-stage knee OA cases were extracted. Cartilage wear patterns were observed by computing joint space width. The three-dimensional joint space width data was translated into a two-dimensional pixel image, which served as the input for a principal polynomial autoencoder developed for non-linear encoding of wear patterns. Virtual healthy twin reconstruction enabled the investigation of the morphology-related risk for OA requiring joint arthroplasty.

**Results:** The polynomial autoencoder revealed 4 dominant, orthogonal components, accounting for 94% of variance in the latent feature space. This could be interpreted as medial (54.8%), bicompartmental (25.2%) and lateral (9.1%) wear. Medial wear was subdivided into anteromedial (11.3%) and posteromedial (10.4%) wear. Pre-diseased limb geometry had a positive predictive value of 0.80 in the prediction of OA incidence (r 0.58, *p* < 0.001).

**Conclusion:** An innovative methodological workflow is presented to correlate cartilage wear patterns with knee joint phenotype and to assess the distinct knee OA risk based on pre-diseased lower limb morphology. Confirming previous research, both alignment and joint geometry are of importance in knee OA disease onset and progression.

## 1 Introduction

Osteoarthritis (OA) globally ranks among the most prevalent disabling diseases, affecting over 500 million people worldwide, which accounts for 7% of the world’s population (Vos et al., 2012; Hunter et al., 2020; Boer et al., 2021). Specifically for an adult USA population, it is estimated that 1 out of 4 inhabitants have some form of arthritis. The OA prevalence is rapidly increasing and is estimated to raise by 50% by the year 2040 [Centers for Disease Control and Prevention (CDC), 2006]. Of the global disease burden for OA, knee OA constitutes 83% (Vos et al., 2012).

However, the precise pathophysiology of OA and drivers for disease progression remain poorly understood (Grässel et al., 2021). Recent literature demonstrates OA being a multi-faceted total joint disease (Boer et al., 2021; Grässel et al., 2021). In the early stage of OA, the cartilage thickness increases as the water fraction raises due to a damaged collagen network. Once the microscopically damaged cartilage fails to counterbalance extrinsic loading, a catabolic reaction initiates superficial articular cartilage loss. Degradation products initiate an inflammatory chain reaction worsening total joint degradation (Grässel et al., 2021). Yet, improved insight in disease onset and progression not exclusively depends on the understanding of these inflammatory pathways. To date, most theoretical approaches of knee OA development and progression assume a synergetic effect of mechanical factors and the systemic milieu. In essences, higher vulnerability in a susceptible environment is assumed to promote disease onset and progression. Inversely, this implies a possible presence of at risk joint mechanics with minimal intra-articular cartilage decay (Sharma et al., 2010).

Recent research strongly emphasizes the link of lower limb alignment and knee joint morphotype on the one hand with the development and progression of knee osteoarthritis on the other hand. Whereas the link between the joint mechanics and OA disease progression is becoming generally accepted, the impact of joint morphology and alignment on the risk of incident knee OA is ambiguously defined. Based on multiple longitudinal observational investigations, varus alignment contributes to incident knee OA with a growing risk for worse varus malalignment. Fewer consensus is observed for valgus alignment (Sharma et al., 2010; Felson et al., 2013; Sharma et al., 2013). From a biomechanical point of view, mild to moderate valgus malalignment is often considered not damaging, as the ground reaction force extending from the center of the foot towards the center of mass passes medially of the center of the knee (Sharma et al., 2010; Sharma et al., 2013; Dell’Isola and Steultjens, 2018). Nevertheless, findings of Felson and colleagues suggest mild to moderate valgus malalignment already promotes incident lateral compartment knee joint OA (Felson et al., 2013).

Few studies have investigated susceptible phenotypes for generalized multicompartment OA, as opposed to unicompartmental disease. In this respect, the most trustworthy method to observe and analyze risk factors for disease onset and progression in a cohort and evaluate mediating factors on the process involves controlled longitudinal follow-up studies. However, in the case of slow progressive and chronic diseases such as OA, the time-dependent character of these experiments limits their discovery power (Murphy, 2021). Furthermore, this multifaceted interaction of joint morphotypes, altered loading conditions and systemic factors results in an extremely heterogenous disease presentation, complicating conceptualization and radiographic classification of knee OA (van der Esch et al., 2014).

Taking into account the co-existence of systemic and local factors, the hypothesis of knee OA being exclusively a unicompartmental disease is more frequently being abandoned. Recent findings of van der Esch and colleagues confirm the combination of a multicompartmental disease process with radiographic features present in the entire joint, on one hand, and a more localized compartmental disease process on the other hand. Based on a bifactor model, joint space narrowing and osteophyte formation are features of both the multicompartmental and the compartmental disease process. As such, osteophyte formation is not useful as a joint overload localizer. Considering both systemic and mechanical factors are involved in the etiology of knee OA, this disease model aligns with the typically observed complex pattern of associations between radiographic features between and within compartments (van der Esch et al., 2014). Typically, the radiographic features describing severity and disease progression are obtained from ordinary X-ray imaging and are subsequently translated into discrete grading systems (e.g., the Kellgren-Lawrence classification) (Kohn et al., 2016). Doing so, a complex three-dimensional problem is simplified to a 2D projection and quantized into a discrete scoring system impeding profound disease phenotyping and classification (Schiphof et al., 2008). Furthermore, the inability to visualize the cartilage layer, negatively impacts the accuracy of the collected data (Favre et al., 2013).

This can be mitigated using volumetric imaging such as Computed Tomography (CT) or Magnetic Resonance Imaging (MRI). Both methods allow for a regional analysis of the joint space width and the detection and description of local erosion (Favre et al., 2013). Usually and for the purpose of comparison between multiple subjects, average cartilage thickness of specific Regions of Interest (ROI) are then obtained. Although being an improvement to standard X-ray measures, this method still fails to a certain extent to comprehensively describe the location and severity of erosion (Favre et al., 2017). Recent studies indeed indicate that the ROI-based methods both under clinical and experimental conditions poorly reflect mild (or early) phases of disease, still demonstrate wide interobserver variation and are very non-linear over the range from mild to advanced disease status (Altman and Gold, 2007; Favre et al., 2013; Kohn et al., 2016; Favre et al., 2017).

To tackle the shortcomings of the above described methods and thus allowing the statistical comparison of groups of knees, Favre and colleagues described a novel method to establish anatomical correspondence among the entire articular surface of the knee joint (i.e., providing an anatomical standardization) (Favre et al., 2013; Favre et al., 2017). As such, cartilage thickness can be measured for any given point on the subchondral layer within a single subject (Favre et al., 2013). Their pattern-based approach considered overall “thickness maps (images)” therefore allows for improved characterization of features that are lost when cartilage thickness is reduced to a few independent mean thickness measures (Favre et al., 2013; Favre et al., 2017).

Progress in computer vision and image analysis now offers efficient methods to establish anatomical correspondence between knee shapes and to describe the joint space to perform statistical comparison and pattern analysis of sets of joint space narrowing maps (i.e., convolutional neural networks following conformal mesh parameterization) (Audenaert et al., 2019a; Nauwelaers et al., 2021). Whereas Favre and colleagues aimed for anatomical correspondence relying on standardized two-dimensional pixel-maps, anatomical correspondence in 3D can be obtained by non-rigid surface registration of a reference template thereby providing a dense set of homologous landmarks amenable to statistical analysis (Williams et al., 2010; Favre et al., 2013; Audenaert et al., 2019a; van Houcke et al., 2020; Peiffer et al., 2022). This permits statistical comparison between and within populations of skeletal shape and related joint space geometry (Williams et al., 2010; Audenaert et al., 2019a; Nauwelaers et al., 2021). Furthermore and by comparison with healthy data, disease severity and progression can be quantified and even a virtual healthy twin can be generated (Fuessinger et al., 2019; Ahmadian et al., 2022).

In the present study, virtual healthy twins are generated from a large cohort of cases presenting with end-grade knee OA, using a validated lower limb shape model (SSM). The latter was constructed, from a healthy cohort of over 600 cases (Audenaert et al., 2019b). Hence, the aim is to return backwards by reconstructing the original alignment and joint morphology prior to OA onset. Doing so, we intend to confirm existing associations and establish new phenotypes at risk for OA onset and progression. In addition to improving the description of cartilage morphology, identifying characteristic wear patterns could provide new insights into the function and degradation process of knee cartilage in relation to the limb alignment and joint morphotype.

## 2 Materials and methods

### 2.1 Sample/data

#### 2.1.1 Osteoarthritis group

**FIGURE 1:**
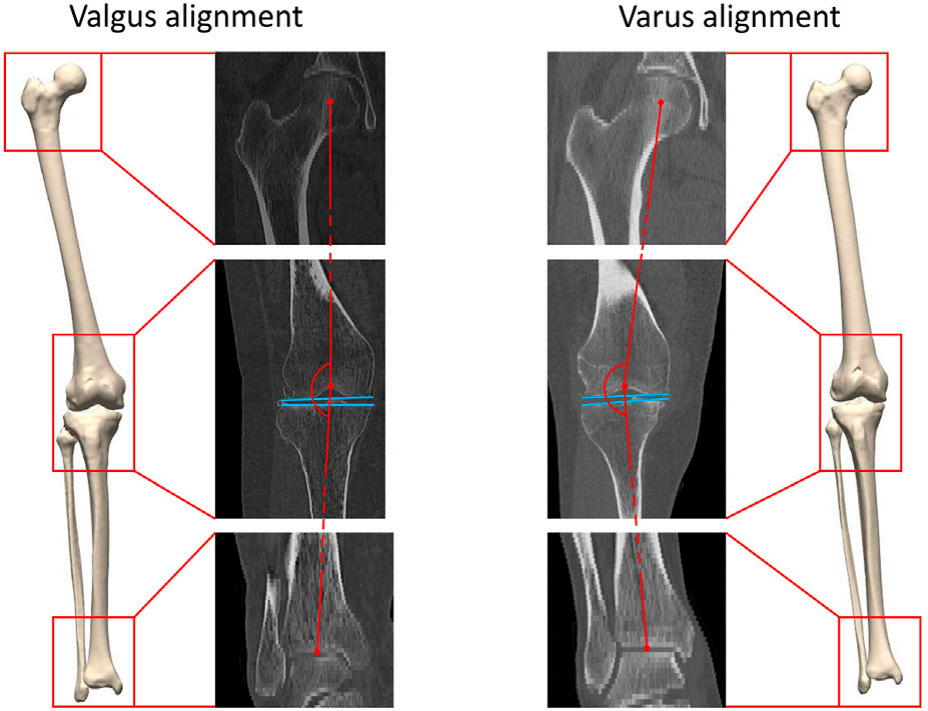
A three-dimensional lower limb model is developed starting from the available CT data. Both valgus alignment (left) and varus alignment (right) are visualized. Typically, valgus alignment (left) is defined as a hip-knee-ankle (HKA) angle smaller than or equaling 177° whereas varus alignment (right) involves a HKA angle equaling or exceeding 183° (Hirschmann et al., 2019). The HKA angle is formed interconnecting the center of the hip, of the knee and of the ankle in the coronal plane, visualized in red. The tangent to the distal femoral bone and to the proximal tibia plateau are visualized in blue.

Digital bony shapes of the proximal and distal femur and the proximal and distal tibia were extracted from a retrospective database of 933 patients (460 females and 473 males), provided in anonymized form by the Medacta company (Medacta International SA, Castel S. Pietro, CH). The patients reported localized knee pain associated with mechanical knee instability at staging time. Diagnostic imaging confirmed different degrees of cartilage defects, femoral osteophytes, and shape abnormalities, mainly at the condylar regions of the distal femur and at the tibial plateau. All patients underwent knee resurfacing or knee replacement surgery by means of a Patient Specific Instrumentation (PSI) between July 2012 and April 2020. For surgical planning purposes, 3D imaging by CT scanning of the lower limb joints was acquired. The image acquisition protocol included CT scans of the knee, hip, and ankle regions. A minimum of 512 × 512 pixels was acquired for each scan. The thickness of a single slice was 2 mm for the hip and ankle joint and 6 mm for the knee joint (Figure 1). All subjects gave written informed consent for data collection and processing before enrollment in the study database. Exclusion criteria were defined as the presence of hip, knee or ankle arthroplasty, the presence of osteosynthesis material, or osteological evidence of former osteosynthesis for trauma or osteotomy reasons as defined by cortical interruptions and the presence of former drilling holes. This study was executed conform the Helsinki guidelines and was approved by the ethics committee of the Ghent University Hospital.

#### 2.1.2 Articulated statistical shape model of the nonarthritic lower limb

For the purpose of reconstruction of a healthy virtual twin, an articulated statistical shape model (SSM) of nonarthritic lower limb cases was used. The Ghent lower limb model is the largest articulated SSM of the lower limb previously reported in the literature, including 622 samples obtained from 311 lower limb CT scans of non-arthritic cases. Additional details about model development and validation are published by Audenaert et al. (Audenaert et al., 2019a). As the SSM was developed based on healthy subjects, osteoarthritis-related deformities are unknown to the model (Sharma et al., 2010; Dell’Isola and Steultjens, 2018).

### 2.2 Healthy virtual twin reconstruction

The virtual healthy twin can be considered the patient-specific original lower limb constitution before the onset of osteoarthritic joint deformation. To generate the healthy twins, the healthy SSM was fitted to a combined femoral head, knee joint and distal tibia. In detail, the following pipeline was used for the reconstruction of the unaffected, pre-disease configuration for each knee OA case.

**FIGURE 2:**
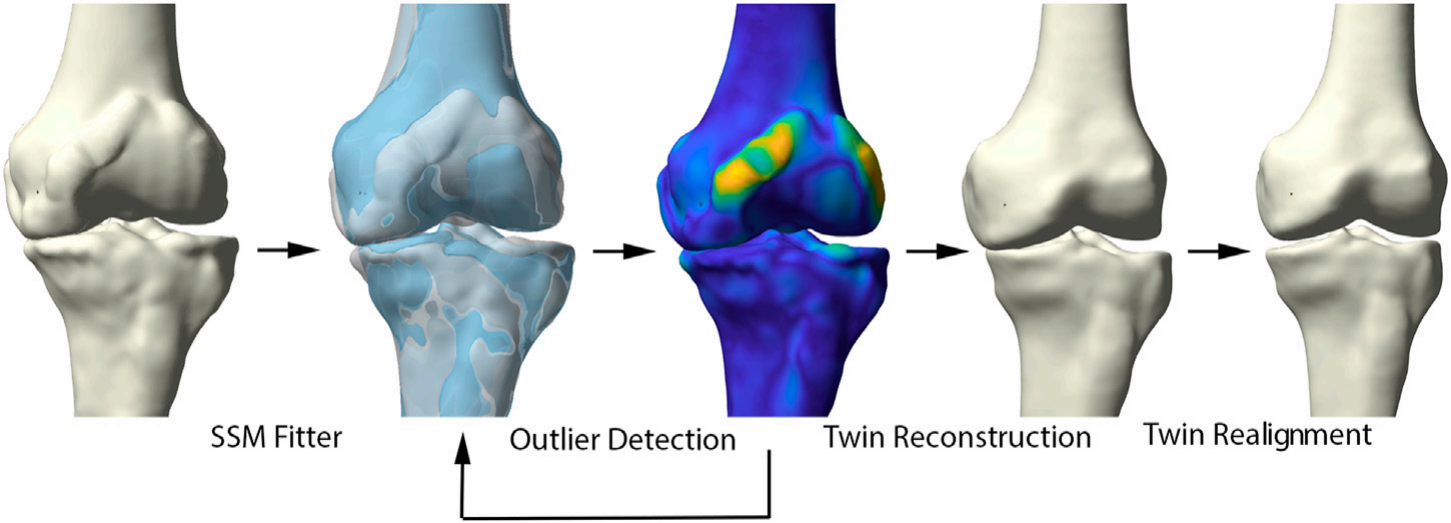
Representation of the healthy virtual twin reconstruction. Left to right: starting from a diseased sample, healthy femur and tibia were fitted to the arthritic knee. Outlier detection aided in osteophyte localization. Following, the healthy statistical shape model was fitted to the diseased sample, excluding disease related morphological changes. The virtual healthy twin was then reassembled based on the healthy, articulated lower limb model.

Healthy virtual twin geometries of respectively femur and tibia were constructed by iteratively fitting the healthy shape model to the patient geometries. The fitting iteratively defined correspondences and excluded outlier candidate pairs, which were assumed to represent local deformations induced by the disease. Outlier vertices were identified by evaluating the distance between each pair of points and defined to deviate more than 2 standard deviations from the mean. Dense correspondences and the deformation are computed in an interleaved fashion until convergence. Following, the healthy reconstructions of femur and tibia were aligned according to the healthy articulated model of the lower limb. The process of reassembly of multiple components according to a control statistical shape model was previously validated by Audenaert et al. (Murphy, 2021). The workflow is illustrated in detail in Figure 2.

**FIGURE 3:**
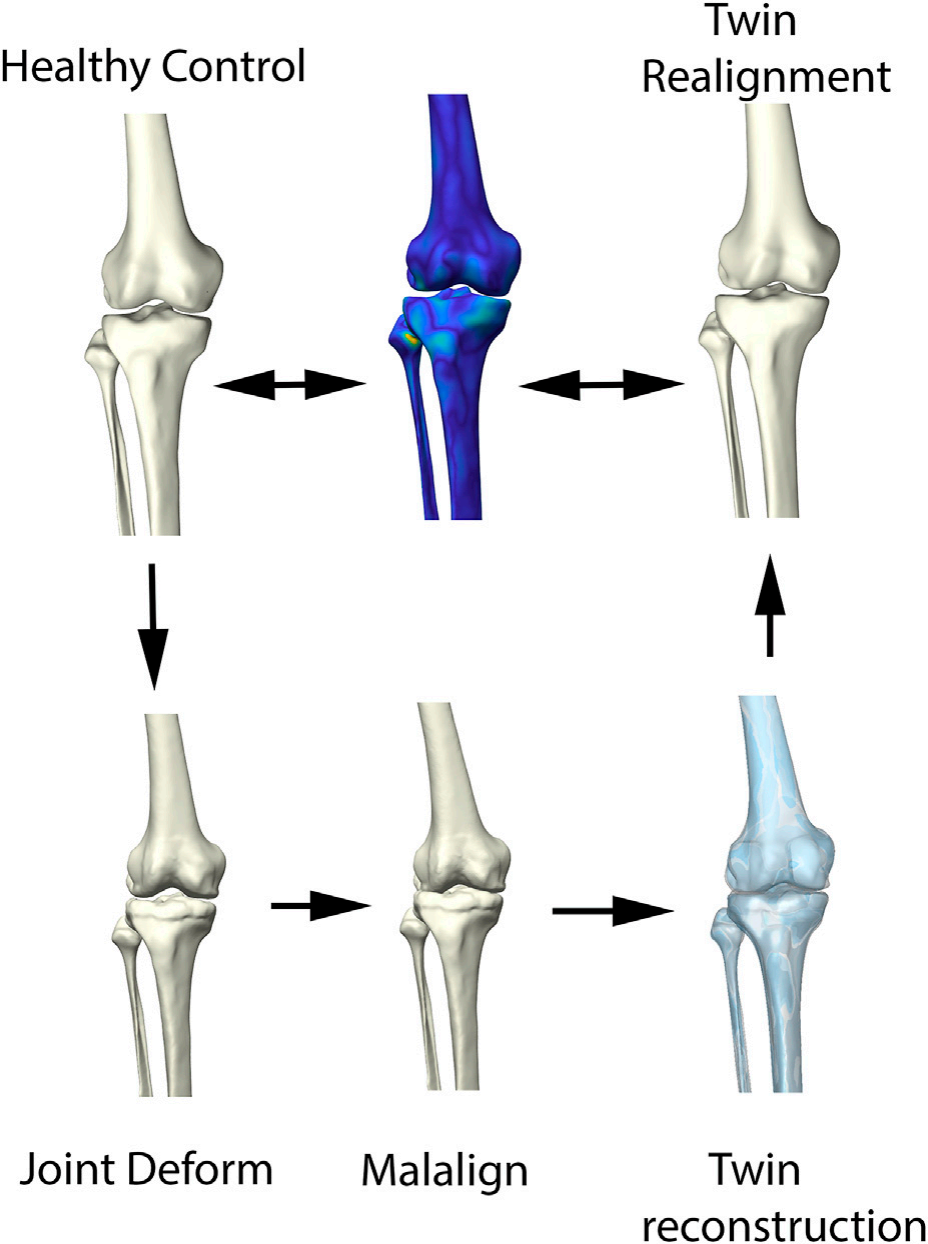
Visual representation of the synthetic validation experiment. Starting from the healthy control subjects, osteophytes are artificially imposed based on available outlier distance maps. Following, misalignment of the femur and tibia was enforced. The virtual twin reconstruction pipeline was then conducted and the virtual, realigned healthy twin was compared with the original healthy control input.

The ability of the above described process to reconstruct a patient’s pre-diseased state was evaluated in a synthetic validation experiment. Healthy cases, not included in the non-arthritic lower limb SSM were randomly provided with outlier deformities around the knee joint, based on distance maps generated from the OA population. Consecutively, misalignment in combination with joint space narrowing was enforced. Following, the reconstruction pipeline was used to reconstruct the native input geometry. The reconstruction error was evaluated by means of the overall root mean square (RMS) reconstruction error and mean absolute angular reconstruction error in the coronal plane (varus -valgus). This process is visualized in Figure 3.

### 2.3 2D Joint space mapping

**FIGURE 4:**
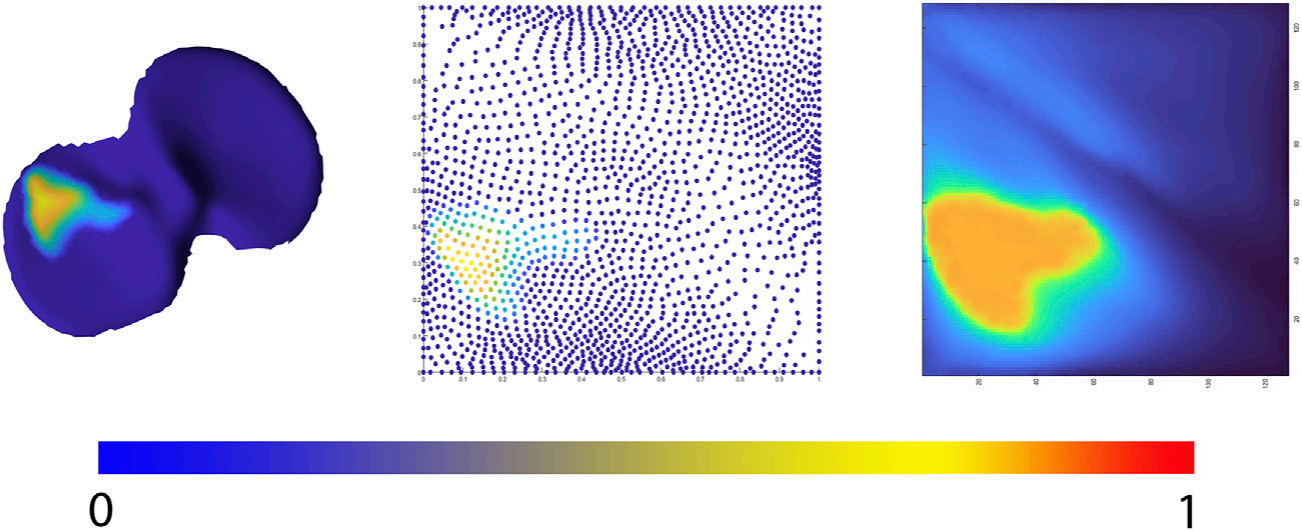
Representation of the 2D joint space mapping workflow. Starting from an anatomically standardized distance map (left) a 2D parameterization (center) is developed. Spline-based interpolation provides a pixel image (right). The continuum from intact cartilage towards complete cartilage erosion is color-coded, ranging respectively from blue = 0 (completely intact cartilage) to red = 1, (complete cartilage erosion).

Allowing statistical comparison of joint space loss between samples, femorotibial joint distance maps were defined, benefitting from the anatomical correspondence established by the shape modelling pipeline. Following, the corresponding distance maps were unwrapped towards a square boundary 2D mesh using a conformal mapping technique. The distance information, visualized in the 2D mesh as colored dots, was subsequently spline-based interpolated to provide a 128 by 128 isotropic pixel image to be used in neural-based learning. Joints space measures were normalized and inverted in such way that a value 1 corresponds with complete cartilage erosion or a joint space width of 0 mm. The workflow is illustrated in Figure 4.

### 2.4 Non-linear encoding of wear patterns: Principal polynomial autoencoder

**FIGURE 5:**
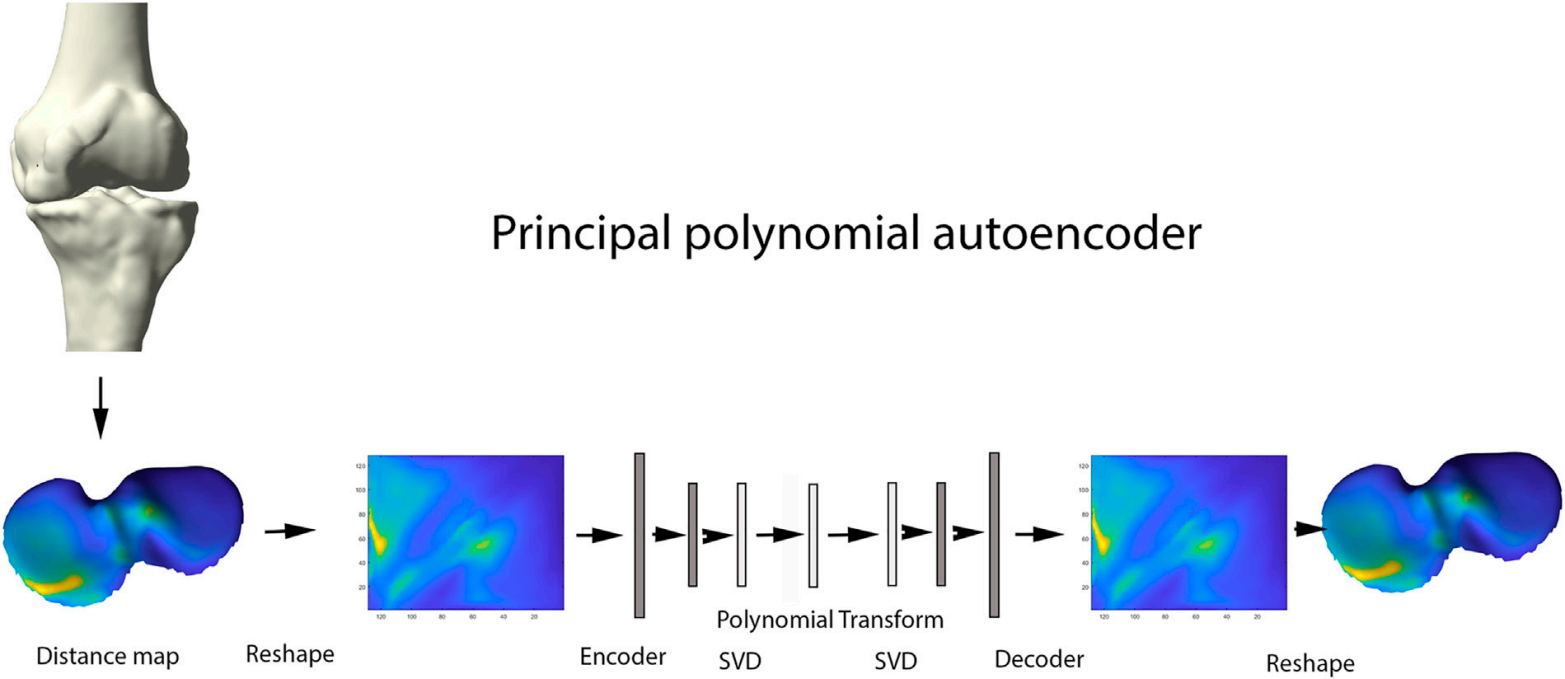
Representation of the principal polynomial autoencoder (PPAE). Anatomically standardized distance maps are encoded to pixel images, serving as input for the autoencoder. Singular value decomposition and consequent polynomial regression replace the last layer of the encoder and the first layer of the decoder.

Principal Component Analysis (PCA) is a widespread statistical technique for dimensionality reduction. Linearly transforming the data in a new coordinate system, variation in the data is captured in multiple principal components. The previously obtained joint space images, however, are inherently non-linear and are as such not amenable for analysis by classical linear techniques including PCA (Nauwelaers et al., 2021). Nevertheless, this type of data is ideal for use in neural-based learning. In this domain, autoencoder networks represent the standard for unsupervised feature learning of nonlinear input data. Here we present a new extension of the technique: the principal polynomial autoencoder (PPAE). The PPAE has the structure of a common AE, except for the bottleneck; i.e. the last layer of the encoder and the first layer of the decoder, which are replaced by a low-rank singular value decomposition and consequent polynomial regression to ensure decorrelated scores (Figure 5).

AE networks have been introduced as a non-linear generalizations of PCA. AE are classified as unsupervised learning methods for pattern identification in complex biological data. The commonly used AE networks consisted of two main parts: an encoder and a decoder. The encoder compresses the data into a small number of variables where the decoder aims to reconstruct the original data from that compact representation. One advantage of using an AE over using popular data compression methods such as PCA, is that linearity is not assumed. As such, non-linear cartilage wear patterns can be captured at different stages of OA progression. An important disadvantage of using AE however, is the resulting variables not necessarily being uncorrelated, opposed to the strictly uncorrelated outcome variables following PCA (Hinton and Salakhutdinov, 1979). Nauwelaers and colleagues effectively combined the power of PCA with the flexibility of deep learning for 3D palatal shape modelling (Nauwelaers et al., 2021). Nonetheless, PCA decomposition is hampered by enforcing complex data in linear independent components, while biological features often present non-linear relations. To overcome the latter, Duquesne et al. introduced principal polynomial regression (PPSA) on a linear feature space such as shape analysis. This technique builds further on Principal Polynomial Analysis (PPA), which itself is an adaptation on PCA enabling to capture non-linear behavior of data. Being very computational expensive, Duquesne and colleagues introduced PCA prior to PPA to reduce the dimensionality in the PPSA technique. Based on this PPSA technique, we extend the concept of singular autoencoder analysis to its non-linear upgrade of principal polynomial analysis on the linear latent feature space to increase specificity and enhanced interpretability of the observations (Duquesne et al., 2022).

Considering an unbalanced incidence between medio-lateral and antero-posterior compartment wear, data augmentation based on left-right and antero-posterior mirroring was conducted prior to the encoding of the original distance plots towards the pixel images, which serve as input for the autoencoder (step 3). This way, 4 times as much training data was offered for neural network training. A simple model architecture was adopted, consisting of two fully connecting layers to encode the input distance images into a compact latent space of 15 dimensions. Model and hyperparameter performance was evaluated by means of the mean squared error metric with L2 and sparsity regularization.

### 2.5 Correlation between wear and morphotype

Following unsupervised feature learning and consequent polynomial parameterization of wear patterns, Uniform Manifold Approximation and Projection (UMAP), was applied to generate a 2-dimensional visual interpretation of the resulting 15-dimensional feature space (Becht et al., 2019; Dorrity et al., 2020). Contrary to PCA, a dimensionality reduction technique based on linear projection of data, UMAP is a non-linear dimension reduction technique. Proven to largely preserve the global structure of the data and maintain high visualization quality, it is often used for data visualization purposes (Becht et al., 2019; Dorrity et al., 2020). Similarly, the combined shape space of healthy twin reconstructions and healthy controls was visualized by this dimensionality reduction method. Canonical correlation analysis is a statistical tool to assess correlation between two sets of variables. Extended from the multiple regression concept, canonical correlation analysis is applied in the presence of multiple intercorrelated outcome variables. Therefore, it was applied to investigate the correlation with alignment and respectively femoral and tibial bony phenotypes, avoiding manual measurements of alignment. Focusing on the presence of correlation, canonical correlation analysis is unable to perform risk assessment. Therefore, the morphology related risk for knee OA requiring joint arthroplasty was evaluated by Linear Discriminant Analysis, benchmarking the geometry of the healthy twins of the arthritic samples against the control population. Linear discriminant analysis (LDA) is a statistical technique that compares to logistic regression. In the case of normally distributed predictor variables, however, it has proven to be a more efficient classifier (Efron, 1975). More specifically, a LDA classifier was trained using the principal component loadings of the shape entries as predictors, outputting a binary scoring for OA requiring joint arthroplasty. The performance of the LDA classifier was than evaluated in a k-fold leave-one-out cross-validation. Shape features were normalized in the Mahalanobis space to decrease the impact of confounding variance of dominant, unrelated features such as size (Audenaert et al., 2019a; Audenaert et al., 2020). The LDA classifier was evaluated in terms of positive predictive value (PPV), sensitivity and specificity.

A negative control experiment was performed using a cohort of healthy controls with artificially introduced deformities and misalignment. The aim of this second validation was to evaluate to what account the twin reconstruction pipeline impacted on the prediction accuracy of the so called native geometry on the associated risk for AO development. As the deformities were induced randomly, there is no expected correlation with the underlying morphotypes. and any correlation observed in this negative control experiment would then be attributable to the reconstruction pipeline and not the native geometry itself.

## 3 Results

### 3.1 Demographics

Out of the 933 subjects from the provided retrospective database, a total of 798 cases (399 females and 399 males) were found to fit the inclusion criteria and were therefore eligible for retrospective assessment. In the female population, a total of 61 cases were excluded for the presence of hip prosthesis (*n* = 40), knee prosthesis (*n* = 5), osteosynthesis material (*n* = 2) or for the existence of segmentation and/or registration errors (*n* = 14). Similarly, combining the number of cases presenting with hip prosthesis (*n* = 42), knee prosthesis (*n* = 5), osteosynthesis material (*n* = 15) or segmentation and/or registration errors (*n* = 12), a total of 74 cases were excluded in the male population. The mean age of the included subjects was 70.5 (±8.2) years. The control population, underpinning the healthy articulated SSM, consisted of 622 non-arthritic limbs obtained from 311 patients (181 males and 130 females). The average age was 68.3 (±11.8) years (Audenaert et al., 2019a).

### 3.2 Synthetic validation experiment

Out of the first validation experiment, the reconstruction error was estimated at an RMS of 0.56 mm (±0.53 mm). The respective bone reconstruction error was 0.64 mm (±0.63 mm) for the femur and 0.53 (±0.47 mm) for the tibia. Errors introduced by the joint realignment were evaluated by comparing the predicted versus original geometries and measured 1.29° (±1.05°) in the coronal plane.

### 3.3 Cartilage wear pattern analysis

**FIGURE 6:**
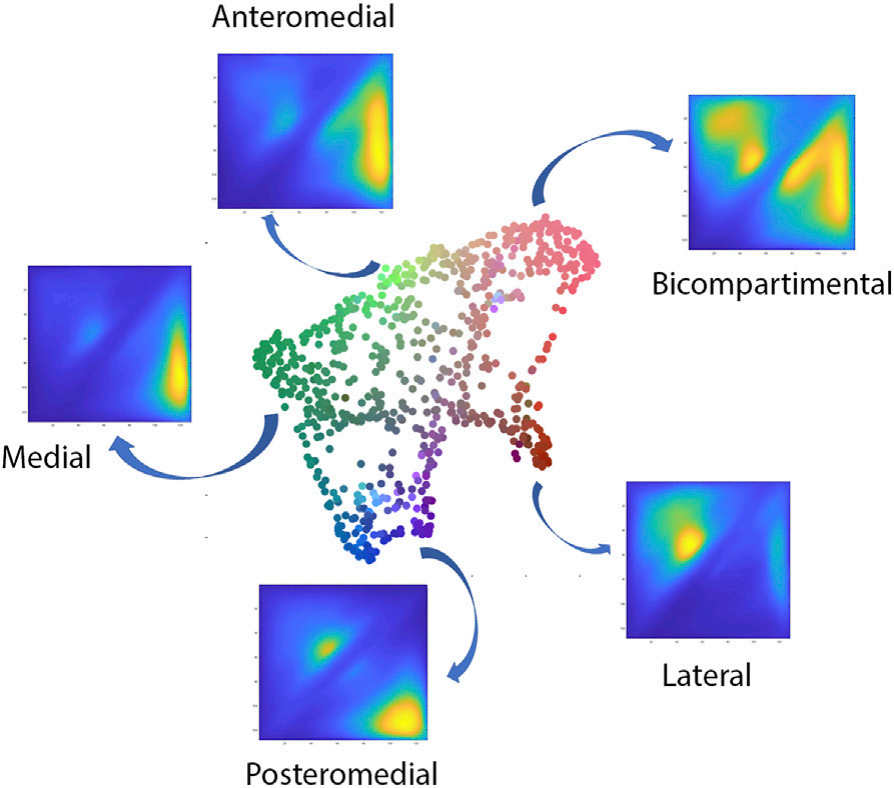
Visual representation of the projected shape space of OA cases with RGB coloring according to the observed value of the first three principal polynomial components.

The polynomial autoencoder revealed 4 dominant, orthogonal components, cumulatively accounting for 94% of variance in the latent feature space. Clinically, the obtained modes or cartilage wear patterns could be interpreted as medial (54.8%), bicompartmental (25.2%) and lateral (9.1%) wear. Two particular wear patterns were present within the polynomial components describing respectively a distinct subtype of medial wear, namely anteromedial (11.3%) versus posteromedial (10.4%) wear, as well as tibial spine impingement (4.5%) as observed in coronal tibiofemoral subluxation. A 2D projection of the polynomial encoder feature space using UMAPS is presented in Figure 6.

When evaluating the correlation of the wear pattern with native limb geometry, significant relations were observed, most prominent with varus valgus-alignment. When visualizing the shape configurations correlating with the observed wear patterns the following observations were made. The primary mode of variation, indicating medial towards bicompartmental wear accounted for nearly half of the variance within the latent space (49.5%). This component correlated significantly with the healthy twin geometry, in particular varus alignment (*r* = 0.4, *p* = 1.43E-19). Varus alignment correlating with this wear type presented with associated lateral bowing, proximal femoral varus, a decreased femoral version and increased tibial internal torsion.

**FIGURE 7:**
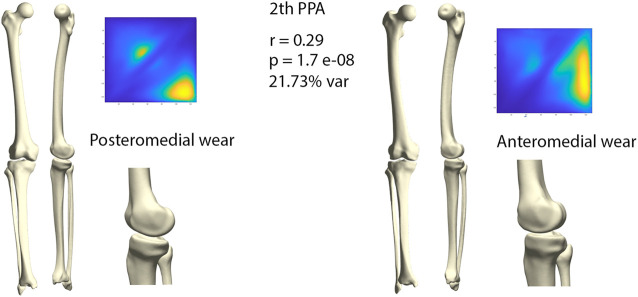
Comparison of limb geometry corresponding with posteromedial (left) and anteromedial (right) cartilage wear. Posteromedial wear is correlated with neutral to varus alignment, increased tibial slope and external tibial torsion. Anteromedial wear is correlated with a constitutional varus, increased femur bowing, decreased femoral torsion and a neutral tibial slope. For visualization purposes, differences obtained following the canonical correlation analysis were amplified with a factor 4.

The second orthogonal component was found to further differentiate medial wear into anteromedial and posteromedial wear (*r* = 0.29, *p* = 1.71E-08) (Figure 7). Limb geometry corresponding with posteromedial wear demonstrated a neutral to varus alignment, increased tibial slope as well as increased external tibial torsion, whereas the limb geometry corresponding with anteromedial wear corresponded mostly with what is clinically considered as a constitutional varus, increased femur bowing, decreased femoral torsion and a neutral tibial slope.

A distinct pattern of lateral wear was captured within the 3^rd^ mode of variance, correlating with valgus alignment. Within this component, 9.12% of wear was described as lateral (*r* = 0.25, *p* = 2.01 Ee-05). Valgus alignment correlating with this wear pattern presented with associated medial bowing, proximal femoral valgus, increased femoral anteversion and increased tibial external torsion.

Finally, the 4^th^ mode of variation described respectively lateral and medial spinal wear or impingement, accounting for 4.53% of feature space variance. This pattern of wear correlated the least with native joint geometry and with the lowest significance value (*r* = 0.19, *p* = 0.013).

### 3.4 Morphology based osteoarthritis risk assessment

**FIGURE 8:**
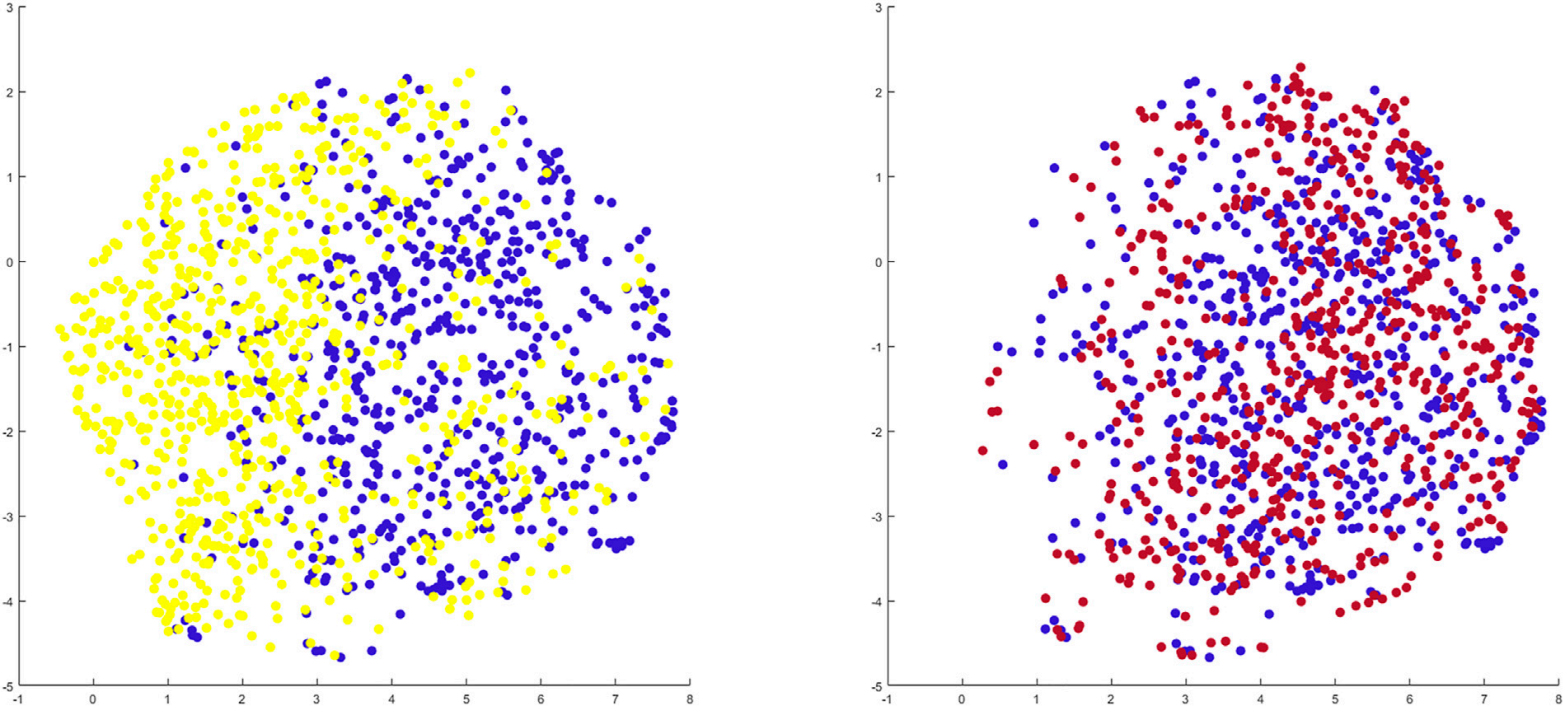
2D representation of multiple shape spaces using UMAPS The depicted shape spaces represent the healthy control population (blue), the virtually reconstructed healthy twins from the diseased subjects (yellow) left panel and the virtually reconstructed healthy twins from the artificially deformed and misaligned healthy control samples (red) right panel. Largely overlapping shape spaces are observed for the heathy control subjects and their virtual reconstruction following artificial deformation whereas distinct phenotypes are delineated between the healthy control samples and the virtual healthy twins, reconstructed out of the arthritic samples.

When benchmarking the geometry of the healthy twins of the arthritic samples against the control population there was a relevant and significant correlation between native geometry and the risk of OA development requiring joint arthroplasty (*r* = 0.58, *p* = 6.31 E-119). When projecting the shape space in 2D (UMPAS) the healthy control group and OA healthy twins, representing the native limb geometry of the currently diseased cases, delineated as distinct phenotypes (Figure 8). Shape features were normalized to minimize the impact of confounding variance of dominant features such as patient size. Limb alignment and shape demonstrated an overall positive predicted value of 0.80 for OA requiring joint arthroplasty development and a negative predictive value of 0.74. The observed sensitivity and specificity equaled respectively 0.81 and 0.73. The sex specific positive predictive values was 0.83 and 0.76 for respectively the male and female sex, whereas the sex specific negative predictive value was 0.81 and 0.72 for again respectively male and female cases.

### 3.5 Negative control experiment

Benchmarking the geometry of the virtual healthy twins, reconstructed out of healthy samples that were artificially deformed and misaligned, against the original healthy control samples, a negligible and non-significant correlation between both was detected (*r* = 0.06, *p* = 0.72). As shape space was projected in 2D (UMAPS), largely overlapping phenotypes were observed (Figure 8).

## 4 Discussion

The understanding of cartilage wear patterns is important to improve insight in knee OA disease onset and progression. In this study, cartilage thickness was assessed in an anatomically standardized manner. As a consequence, the results aid in the understanding of spatial variation in cartilage degeneration in knee OA. Furthermore, assessing the correlation between limb morphology and the risk for knee OA requiring joint arthroplasty assists in the identification of morphometric risk factors potentially affecting the course of the disease.

In the present study, unsupervised learning was applied on a large cohort of degenerative knee joints to asses distinct patterns in cartilage wear and establish the relation with limb alignment and local joint geometry. The polynomial autoencoder revealed medial and lateral wear to be related to respectively varus and valgus malalignment, confirming previously published findings (Sharma et al., 2010; Sharma et al., 2013; Dell’Isola and Steultjens, 2018; Siddiqi et al., 2022). However, Siddiqi and colleagues assumed coronal plane malalignment being rather a response than a cause of knee OA. In contrast, we found it to be present already in a pre-diseased state (Siddiqi et al., 2022). As such, we consider it to be part of an arthritic phenotype rather than being uniquely a result of knee OA.

Within the group of medial cartilage wear, the polynomial autoencoder revealed a further differentiation into anteromedial and posteromedial wear. Whereas anteromedial wear appeared to correspond with the classic description of constitutional varus, posteromedial wear demonstrated a rather neutral to slight varus alignment in combination with a more pronounced posterior tibial slope. Multiple authors linked greater posterior tibial slope to an increased ACL rupture risk (Lansdown and Ma, 2018; Kim et al., 2019; Mortazavi and Vosoughi, 2022). As the femoral condyle translation increases, the ACL is forced to elongate and the tension placed on the ligament intensifies (Lansdown and Ma, 2018). For ACL deficient knees, the wear pattern was suggested to be distinct and posteromedial as opposed to anteromedial wear in ACL intact varus deformity. Rout and colleagues confirmed these findings by describing a shift from anteromedial wear in ACL intact knees towards progressively more posteromedial wear for increasing ACL damage. These findings suggested ACL deficiency to be a causative factor for posteromedial cartilage wear as it allows greater anteroposterior movements in the tibiofemoral joint (Rout et al., 2013). Having identified a specific phenotype related to posteromedial cartilage wear, our findings were supportive to previous clinical observations. However lacking information about the soft tissue status, we cannot differentiate whether posteromedial wear is solely based on a specific phenotype that additionally increases the ACL rupture risk or whether it develops following ACL rupture, as the specific knee joint morphology makes the subject susceptible for ACL rupture.

Observing the correlation between limb morphology and the risk for knee OA requiring joint arthroplasty, a negative control experiment revealed largely overlapping shape spaces of both the virtually reconstructed healthy twins of the artificially deformed healthy subjects and the healthy control group. As such, the perceived correlation between the predicted native joint geometry and the risk for knee OA requiring arthroplasty is not affected by the reconstruction pipeline. To our knowledge, we are the first to enable distinct knee OA risk assessment based on knee joint morphology in the absence of disease-induced abnormalities. These findings enable accurate and early identification of subjects at risk and so enlarges the number of joint preserving treatment possibilities.

One of the nowadays routinely used preventive treatment options, is joint preserving knee osteotomy surgery (He et al., 2021). As coronal plane malalignment impacts knee joint OA progression, restoring the mechanical axis by joint realignment positively impacts disease advancement (Sharma et al., 2010; Sharma et al., 2013; He et al., 2021). Following osteotomy surgery, knee joint loading is redistributed and knee joint contact pressures are altered (He et al., 2021). We believe that the observed correlation between native joint geometry and the risk for incident knee OA requiring joint arthroplasty enables identification of young, healthy subjects at risk. As a result, those with a high risk can then be treated with joint realignment surgery in a pre-diseased state. Aiming to detect the drivers in joint development and morphology, the genetic background becomes of importance. Although rapidly evolving, genetic research for orthopedic purposes is a relatively recent research domain. Regarding the hip joint for example, Evangelou et al. performed a Genome Wide Association Study (GWAS) meta-analysis on cartilage thickness of the hip joint to detect genetic polymorphisms (Evangelou et al., 2014; Boer et al., 2021). Similar research for detection of knee OA drivers is currently lacking. However, the identification of genetic polymorphisms that lead to knee OA promoting phenotypes can aid in early detection of at risk subjects.

Currently, the identification of subjects at risk is hampered by the inability to detect early changes in cartilage thickness based on the available OA scoring and classification systems. The amount of cartilage degeneration is typically graded using discrete scoring systems in multiple artificially separated regions of interest (Sharma et al., 2010; Sharma et al., 2013; Favre et al., 2017). Aiming to avoid both artificial separating the cartilage layer and grading the amount of degeneration by means of discrete scoring systems, our data collection relies on anatomically standardized cartilage thickness measurements. Similarly, Favre and colleagues described the use of anatomical cartilage thickness maps (Favre et al., 2017; Favre et al., 2021). Williams et al., on the other hand, established anatomical correspondence relying on SSM (Williams et al., 2010). In general, establishing anatomical correspondence among knees for the entire articular surface (i.e., providing an anatomical standardization) allows statistical comparison of groups of knees. Pattern-based approaches considering overall “thickness maps (images)” can then allow for the characterization of features that are lost when cartilage thickness is reduced to a few independent mean thickness measures (Favre et al., 2013). Population-wide analysis allows documenting disease progression and response to therapy assessments (Williams et al., 2010). In the present work we leverage this approach towards unsupervised learning applications and variance models.

### 4.1 Strengths

Some of the major strengths of this research lies in the innovative methodology. First, reconstructing the original alignment and the joint morphology offers the unique advantage to return to a pre-diseased state.

Second, we present the concept of converting anatomically corresponding color-coded distance maps into pixel images for use in neural-based learning. As biomechanical information is often represented using colormaps, the presented workflow on color-based images for use in neural learning can be extrapolated towards numerous biomechanical research applications. For example, estimation of articular joint contact stresses by means of FEA or DEA typically provides a similar color-coded output (van Houcke et al., 2020).

Finally, conclusions regarding both the phenotype-based distinct knee OA risk and the correlation between cartilage wear patterns and limb morphology were based on a substantial research population of 1420 cases.

### 4.2 Limitations

Besides the strengths, we have to admit some limitations. As we present here a proof of concept for cartilage wear pattern recognition built on neural-based unsupervised learning, we developed a rather straightforward autoencoder consisting of two fully connecting layers. Extending this approach for other, possibly more complex, biomechanical problems as described above, convolutional neural networks might are more appropriate to handle pixel images.

Furthermore, the main focus of the present work lies on the assessment of cartilage wear patterns and on finding correlations between the development of knee OA requiring joint arthroplasty and knee phenotypes. Although the extraction of information about osteophyte formation is possible from the available dataset, this was not evaluated. We opted to focus on joint space narrowing, especially as osteophyte formation is triggered by both local and systemic factors according to the hypothesis of total joint involvement. Thus, osteophyte formation is not necessarily localized close to cartilage erosion but can be seen in the complete joint. Osteophyte formation at the intercondylar notch has been related to increased stress and local wear in case of malalignment and is considered an early sign of OA (Sasho et al., 2017). More detailed research regarding variations in intercondylar notch osteophyte formation is needed, especially since the polynomial autoencoder revealed the 4^th^ orthogonal component to describe medial and lateral notch impingement.

Lastly, although our findings are based on a substantial dataset consisting of both a large number of diseased subjects and a healthy control group, an important limitation of our findings relates to the amount of available clinical information of the patients investigated. For example, information regarding soft tissue status, and in particular the ACL, is absent. Similar and although known to contribute to knee OA disease progression data about Body Mass Index (BMI) and lifestyle-related knee joint loading is lacking (Blazek et al., 2014). Furthermore, a possible selection bias is introduced by excluding the cases present with hip and/or ankle arthroplasty, excluding cases probably suffering OA driven by systemic factors rather than by knee-specific risk factors. However, aiming to establish phenotypes at risk for development of end-stage knee OA, identification of knee-specific risk factors is prioritized in this study rather than identification of systemic risk factors. Lastly, as the results are based on a single homogenous population of Western European Descent, the extent of which findings can be extrapolated to other populations is unknown. The complex interaction between genes, culture and the environment results in a population-based variation, with several studies showing that the appropriate evaluation of this variation requires specific standards for each population (Rissech et al., 2013; San-Millán et al., 2017). Nevertheless, in general we expect our results to be representative by extension for a Western European population.

## 5 Conclusion

In the present study, we developed an innovative methodological workflow to correlate cartilage wear patterns with knee joint phenotype and to assess the distinct knee OA risk based on pre-diseased samples. Our findings confirm previous studies suggesting that both alignment and joint geometry are highly and significantly correlated with the risk of OA onset and progression. Further, particular morphological phenotypes correlate with distinct cartilage wear patterns.

## Data Availability

The data analyzed in this study is subject to the following licenses/restrictions: Digital bony shapes of the proximal and distal femur and the proximal and distal tibia were extracted from a retrospective database of 933 patients (460 females and 473 males), provided in anonymized form by the Medacta company (Medacta International SA, Castel S. Pietro, CH). Requests to access these datasets should be directed to Info@Medacta.ch.
